# Supplementary figures and images for: Pilot randomized clinical trial of virtual reality pain management during adult burn dressing changes: Lessons learned
Source: PLOS Digit Health. 2023 Sep 25;2(9):e0000231. doi: 10.1371/journal.pdig.0000231 (PMC10519584; doi:10.1371/journal.pdig.0000231)

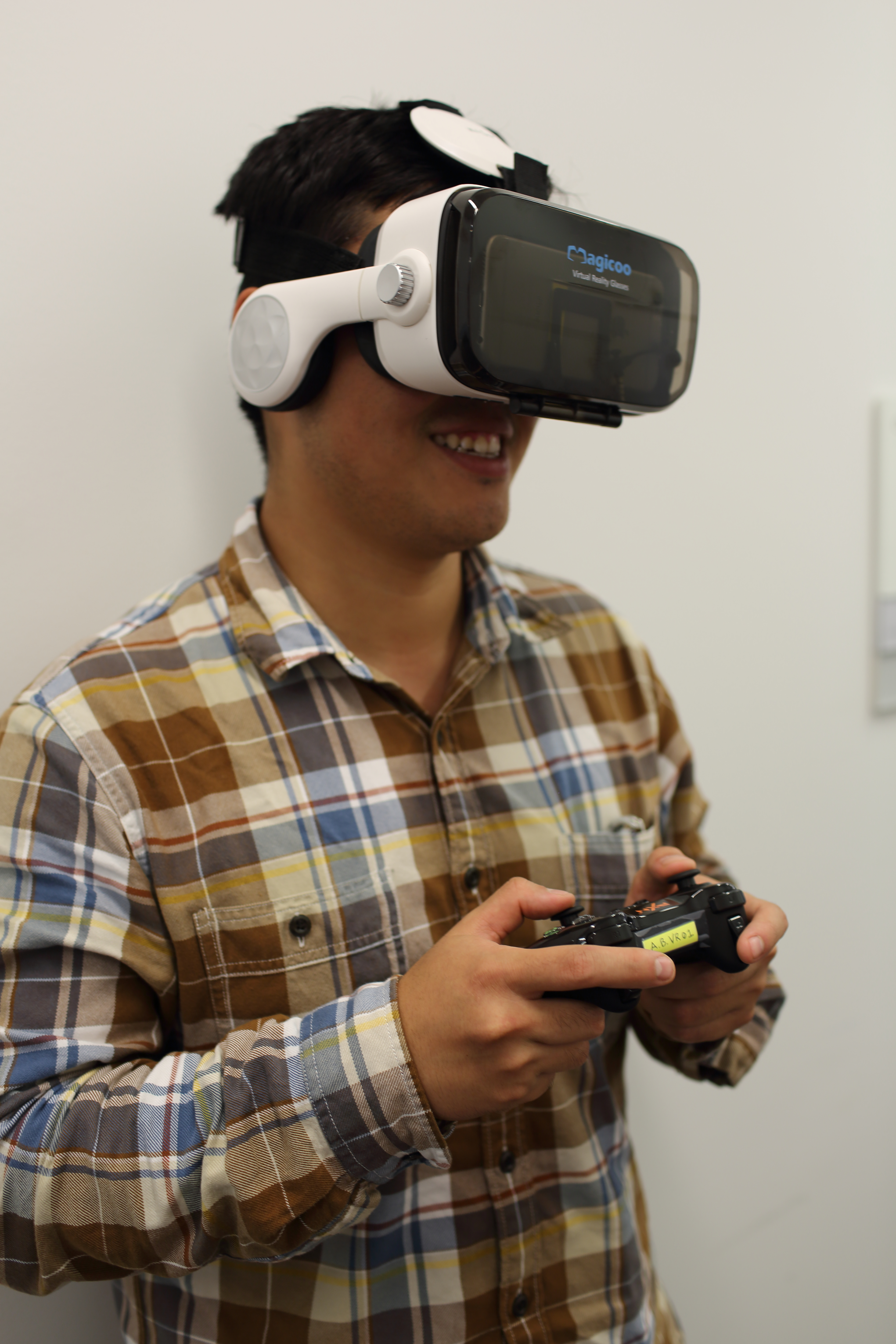

Supplement: S1 Fig — (TIF) [file pdig.0000231.s002.tif]

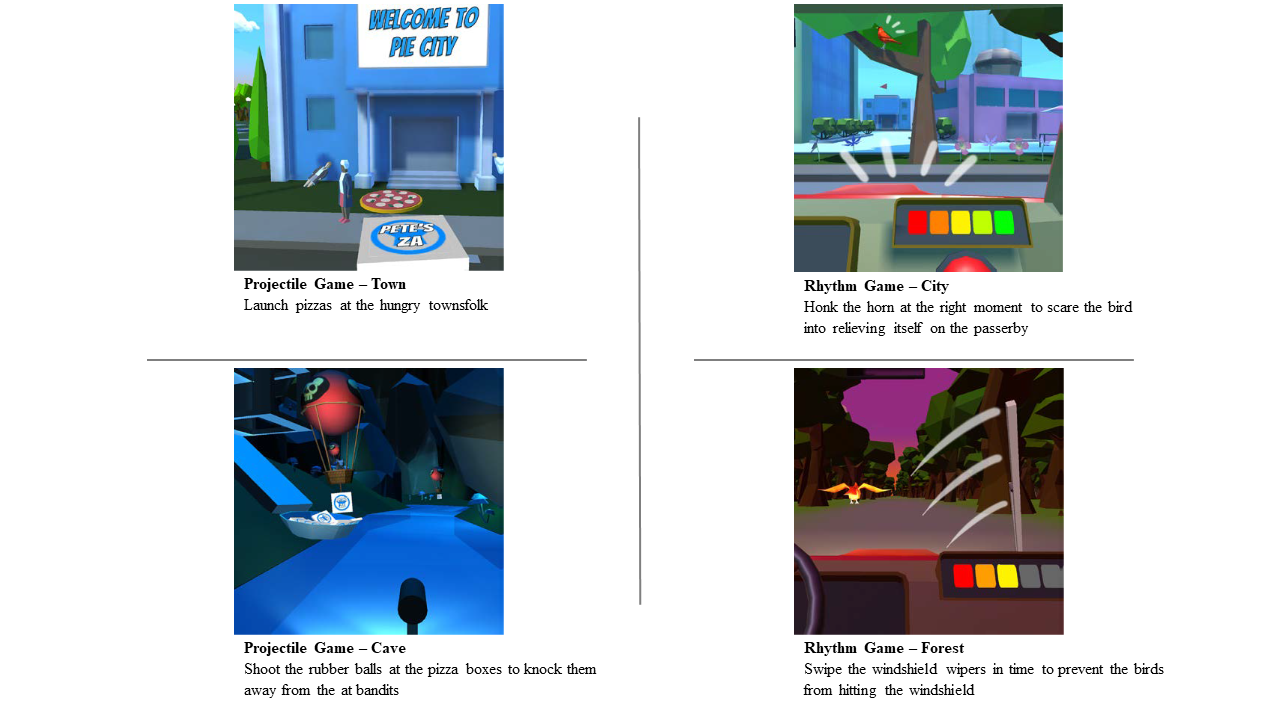

Supplement: S2 Fig — (TIF) [file pdig.0000231.s003.tif]
